# Tribological and Thermo-Mechanical Properties of TiO_2_ Nanodot-Decorated Ti_3_C_2_/Epoxy Nanocomposites

**DOI:** 10.3390/ma14102509

**Published:** 2021-05-12

**Authors:** Yalin Zhang, Xuzhao He, Miao Cao, Xiaojun Shen, Yaru Yang, Jie Yi, Jipeng Guan, Jianxiang Shen, Man Xi, Yuanjie Zhang, Bolin Tang

**Affiliations:** 1 Key Laboratory of Yarn Materials Forming and Composite Processing Technology of Zhejiang Province, Jiaxing University, Jiaxing 314001, China; zhangyalin@zjxu.edu.cn (Y.Z.); 00007889@zjxu.edu.cn (M.C.); sxj908@zjxu.edu.cn (X.S.); yyr0515@zjxu.edu.cn (Y.Y.); yijscu@zjxu.edu.cn (J.Y.); guanjipeng@zjxu.edu.cn (J.G.); shenjx@zjxu.edu.cn (J.S.); xixi123456@zjxu.edu.cn (M.X.); 00169733@zjxu.edu.cn (Y.Z.); 2 Nanotechnology Research Institute, Jiaxing University, Jiaxing 314001, China; 3 State Key Laboratory of Silicon Materials, School of Materials Science and Engineering, Zhejiang University, Hangzhou 310027, China; hf@zju.edu.cn

**Keywords:** Ti_3_C_2_ Mxexe, epoxy, polymer-matrix composites, tribological, thermo-mechanical

## Abstract

The micromorphology of fillers plays an important role in tribological and mechanical properties of polymer matrices. In this work, a TiO_2_-decorated Ti_2_C_3_ (TiO_2_/Ti_3_C_2_) composite particle with unique micro-nano morphology was engineered to improve the tribological and thermo-mechanical properties of epoxy resin. The TiO_2_/Ti_3_C_2_ were synthesized by hydrothermal growth of TiO_2_ nanodots onto the surface of accordion-like Ti_3_C_2_ microparticles, and three different decoration degrees (low, medium, high density) of TiO_2_/Ti_3_C_2_ were prepared by regulating the concentration of TiO_2_ precursor solution. Tribological test results indicated that the incorporation of TiO_2_/Ti_3_C_2_ can effectively improve the wear rate of epoxy resin. Among them, the medium density TiO_2_/Ti_3_C_2_/epoxy nanocomposites gained a minimum wear rate. This may be ascribed by the moderate TiO_2_ nanodot protuberances on the Ti_3_C_2_ surface induced a strong mechanical interlock effect between medium-density TiO_2_/Ti_3_C_2_ and the epoxy matrix, which can bear a higher normal shear stress during sliding friction. The morphologies of worn surfaces and wear debris revealed that the wear form was gradually transformed from fatigue wear in neat epoxy to abrasive wear in TiO_2_/Ti_3_C_2_/epoxy nanocomposites. Moreover, the results of thermo-mechanical property indicated that incorporation of TiO_2_/Ti_3_C_2_ also effectively improved the storage modulus and glass transition temperature of epoxy resin.

## 1. Introduction

Epoxy resin is a kind of typical thermosetting polymer that possesses high mechanical strength, low shrinkage rate, excellent chemical stability, high adhesive strength, etc., and has been widely used in aerospace, petrochemical engineering, automobile components and other fields [1,2,3,4]. However, the intrinsic high brittleness, low resistance to thermal shock, and poor wear resistance of epoxy resin, originating from its highly crosslinked network structure, seriously restrict its practical application and development in many industrial fields, especially in the antifriction and high-frequency vibrating components and parts of machinery [5,6,7]. Therefore, it is quite necessary to improve wear resistance and dynamic mechanical properties of epoxy resin.

To date, the physical blending with fillers is considered as one of the most commonly used ways to improve the multifunctional performance of polymer, especially wear resistance [8,9,10]. In this manner, the high anti-friction feature of fillers can be integrated into polymer matrix composites, thereby improving their wear resisting property. Previous studies have demonstrated that many kinds of wear resistant fillers, such as ZnO [11], PTFE [12], graphite [13], graphene oxide [14], and WS_2_ [15], can effectively improve the wear resistance of epoxy resin, and the particle size of fillers could influence significantly the wear rate of epoxy resin [16]. Shi et al. [17] further found that the nano-sized TiO_2_ particles imparted a better wear resisting property to the epoxy resin when compared to the micro sized TiO_2_ fillers, mainly ascribing to the stronger interfacial interaction between nano-TiO_2_ and epoxy matrix. Therefore, it can be speculated that the interfacial interaction of filler-polymer matrix plays an important role in the wear resisting property of polymer composites, as well as other properties.

The micromorphology of micro/nano particles is one of the key factors that influence the interfacial interaction of particle-polymer. Ramezanzadeh et al. [18] reported that the different micromorphology of iron oxide particles could induce the distinguishing interfacial interaction between iron oxide and epoxy, thereby resulting in the different mechanical properties. In recent years, some publications have indicated that the micromorphology of fillers could influence the tribological and/or other properties of polymer [19,20]. Even, the composite particles with unique micromorphology had been synthesized for improving the tribological property of polymer [21,22]. However, how to design the micromorphology of fillers for imparting the optimal tribological and mechanical property to polymer matrix still remains a challenge.

**Scheme 1:**
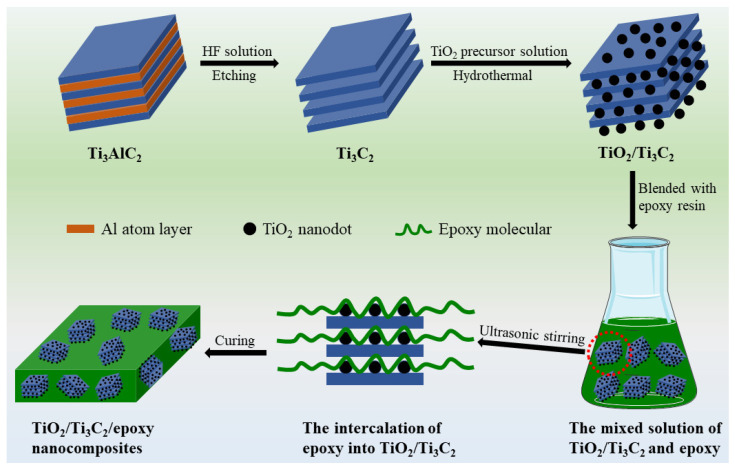
Illustration for preparation of TiO_2_/Ti_3_C_2_/epoxy nanocomposites.

Inspired by the micro/nano composite structure from previous reports [23,24], we plan to design and synthesize a composite particle with unique micro/nano structure by integrating nano-structured particles into micro-structured particles. Herein, Ti_3_C_2_ MXene, a new kind of 2D transition metal carbides firstly discovered by Gogotsi et al. [25] in 2011, was selected as the micro-structure particles, and the TiO_2_ was chosen as the nano-structured particles. It has been widely reported that both Ti_3_C_2_ and TiO_2_ particles can improve the tribological and/or mechanical properties of epoxy and other polymers [26,27,28,29,30]. In this work, we attempted to synthesize the TiO_2_ decorated Ti_3_C_2_ (TiO_2_/Ti_3_C_2_) composite particles that possessed the unique micro/nano structure through hydrothermally growth of TiO_2_ nanodots on Ti_3_C_2_ microparticles, to improve the tribological and thermo-mechanical properties of epoxy resin. After removal of Al atom layers of Ti_3_AlC_2_ by HF, the accordion-like Ti_3_C_2_ microparticles were prepared. Subsequently, TiO_2_ nanodots were hydrothermally grown on the Ti_3_C_2_ surface to construct the micro/nano structured TiO_2_/Ti_3_C_2_, and the TiO_2_/Ti_3_C_2_/epoxy nanocomposites were fabricated by ultrasound-assisted blending of epoxy with TiO_2_/Ti_3_C_2_ (Scheme 1). The micromorphology of different decoration degrees of TiO_2_/Ti_3_C_2_ composite particles and the fracture surface of TiO_2_/Ti_3_C_2_/epoxy nanocomposites were investigated, and the tribological and thermo-mechanical properties of TiO_2_/Ti_3_C_2_/epoxy nanocomposites were tested. Moreover, the underlying wear mechanism of TiO_2_/Ti_3_C_2_/epoxy nanocomposites was analyzed.

## 2. Materials and Methods

### 2.1. Materials

Ti_3_AlC_2_ powders (99.5%, 400 mesh) were purchased from Kaifa Special Ceramic Technology Co., Ltd. (Beijing, China). Tetrabutyl titanate (TBOT), hydrofluoric acid (HF, 40%) and absolute ethyl alcohol (AR) were supplied by Sinopharm Chemical Reagent Co., Ltd. (Shanghai, China). Epoxy resin (E-51), methylhexahydrophthalic anhydride (MHHPA) and tetraethyl ammonium bromide (TEAB) were provided by Haining Hailong Chemical Co., Ltd. (Jiaxing, China).

### 2.2. Preparation of Ti_3_C_2_ Microparticles

Ti_3_AlC_2_ powders were added into HF solution (40%) at a ratio of 0.1 g per milliliter. After stirring vigorously for 12 h at room temperature (RT), the sediments were centrifugally collected at a speed of 4000 rpm, followed by washed with deionized water until the pH value was approximate to neutrality. With the wet powders were dried for 12 h at 60 °C in a vacuum drying oven, the Ti_3_C_2_ microparticles were obtained.

### 2.3. Preparation of TiO_2_ Decorated Ti_3_C_2_ (TiO_2_/Ti_3_C_2_) Composite Particles

Ti_3_C_2_ microparticles (1 g) were added into a mixed solution of deionized water and absolute ethyl alcohol (30 mL, volume = 1:1) to form a Ti_3_C_2_ dispersion solution. Simultaneously, the desired molar mass of TBOT (0.25, 0.5, and 1.0 mol) were added into 45 mL of absolute ethyl alcohol to prepare the TiO_2_ precursor solution. Afterwards, the TiO_2_ precursor solution was added dropwise into the Ti_3_C_2_ dispersion solution with stirring vigorously, followed by transferred into a 100 mL of Teflon-lined autoclave and kept at 180 °C for 24 h. After cooled to RT, the sediments were centrifugally collected at a speed of 4000 rpm, and dried at 60 °C for 12 h in a vacuum drying oven, the TiO_2_/Ti_3_C_2_ composite particles were obtained. According to the molar mass of TBOT, the obtained TiO_2_/Ti_3_C_2_ were named as 0.25 M, 0.5 M, and 1.0 M TiO_2_/Ti_3_C_2_.

### 2.4. Preparation of TiO_2_/Ti_3_C_2_/Epoxy Nanocomposites

Firstly, the desired mass fraction of TiO_2_/Ti_3_C_2_ were ultrasonically dispersed into absolute ethyl alcohol, followed by epoxy resin was mixed with TiO_2/_Ti_3_C_2_ dispersion solution for 1 h under ultrasound. With stirring vigorously, the mixed solution of TiO_2_/Ti_3_C_2_ and epoxy was reduced pressure distilled at 60 °C until the absolute ethyl alcohol was fully evaporated. The MHHPA containing TEAB was then added into the mixture of TiO_2_/Ti_3_C_2_ and epoxy, followed by stirring for 1 h at RT in a vacuum. The TiO_2_/Ti_3_C_2_/epoxy mixture was subsequently poured into stainless steel mold and cured for 2 h at 120 °C, the TiO_2_/Ti_3_C_2_/epoxy nanocomposites were finally obtained. Herein, the mass ratio of epoxy resin, MHHPA and TEAB was set to 100:80:0.3, and the mass fraction of TiO_2_/Ti_3_C_2_ (relative to nanocomposite) was set to 0.25%, 0.5%, 0.75%, and 1%.

### 2.5. Characterization and Measurement

The surface morphologies of the samples were investigated by field-emission scanning electron microscope (FE-SEM, Hitachi S4800, Tokyo, Japan). The phase composition of the samples was analyzed by X-ray diffractometer (XRD, Bruker D8, Karlsruhe, Germany). Fourier transform infrared (FTIR) spectra of Ti_3_AlC_2_, Ti_3_C_2_ and TiO_2_/Ti_3_C_2_ were recorded at room temperature by an infrared spectrometer (Nicolet 5700, Madison, WI, USA) with the range from 400 cm^−1^ to 4000 cm^−1^. The densities of TiO_2_/Ti_3_C_2_/epoxy nanocomposites were measured with a density tester (GP-300E, Guanwei Instrument, Shanghai, China), and the hardness of TiO_2_/Ti_3_C_2_/epoxy nanocomposites were examined by Vickers microhardness tester at a load of 0.5 N and loading time of 15 s (HV-1000, Shangcai Instrument, Shanghai, China). For the testing of tribological property, the TiO_2_/Ti_3_C_2_/epoxy nanocomposites were cut into strip-shaped specimens (16 mm × 4 mm × 4 mm), and specimen surfaces were polished with 1200 mesh SiC abrasive paper. After alcohol washed and dried at 60 °C, the tribological testing of specimens was performed by a vertical universal friction and wear testing machine (MM-W18, Shijin Co. Ltd., Jinan, China) at a rotating speed of 780 rpm with the load of 4 N and 8 N for 1 h duration under dry friction condition. Six replications were carried out for each nanocomposite. The friction coefficients were evaluated as the computer-calculated average value. The average wear rates were calculated according to the formula: W = V/(F × L), where the W, V, F, and L are the wear rate, wear volume, applied load, and sliding distance, respectively [31]. Thermo-mechanical properties were measured using DMA 850, (TA Instruments, Newcastle, DE, USA). The TiO_2_/Ti_3_C_2_/epoxy nanocomposites were cut into strip-shaped specimens (30 mm × 10 mm × 4 mm) to match with machine. A temperature scan was conducted from 40 to 190 °C with a heating rate of 5 °C/min at a frequency of 1 Hz and a strain of 0.05% in a single cantilever mode. The storage modulus and tan δ (the ratio of loss modulus to storage modulus) were obtained from dynamic mechanical analysis, and the glass transition temperature (Tg) was gained from the peak value of tan δ.

## 3. Results

### 3.1. Surface Morphology of TiO_2_/Ti_3_C_2_ Microparticles

**Figure 1:**
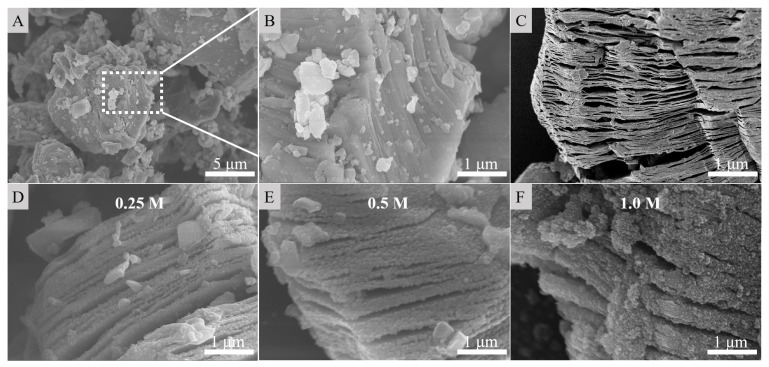
SEM images of Ti_3_AlC_2_ (**A**,**B**), Ti_3_C_2_ (**C**) and TiO_2_/Ti_3_C_2_ synthesized by different concentration of TiO_2_ precursor solution ((**D**) 0.25 mol; (**E**) 0.5 mol; (**F**) 1.0 mol).

The Ti_3_C_2_ microparticles were prepared by HF etching, and the TiO_2_/Ti_3_C_2_ composite particles were synthesized via hydrothermal method. As shown in Figure 1A, the original Ti_3_AlC_2_ microparticles exhibit irregular morphology, with an approximate size of 10 μm, and the selected area magnified image (Figure 1B) clearly displays the tightly stacked layer structure of Ti_3_AlC_2_ microparticles. After etched with HF solution, lots of interlayered gaps are produced in etched Ti_3_AlC_2_ microparticles (Figure 1C), implying the removal of Al atom layers in Ti_3_AlC_2_ and the possible formation of Ti_3_C_2_ microparticles [32]. After hydrothermally reacted with TiO_2_ precursor solution, the surface of Ti_3_C_2_ is covered with large amounts of nanodots (Figure 1D–F), which maybe suggests that the TiO_2_ are successfully grown on the surface of Ti_3_C_2_. It can be seen that the density of nanodots on Ti_3_C_2_ surface is gradually increase with increasing the molar mass of TOBT, especially, the obvious aggregated nanodots are found on the surface of Ti_3_C_2_ when the molar mass of TOBT reaches 1.0 mol.

### 3.2. XRD and FTIR Analysis of TiO_2_/Ti_3_C_2_ Composite Particles

**Figure 2:**
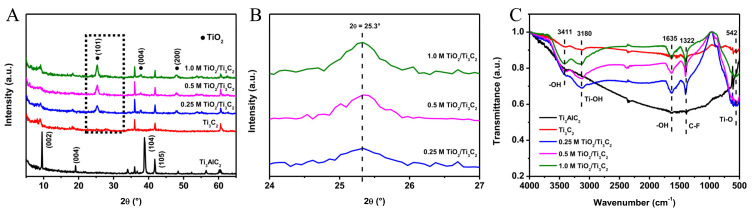
XRD patterns (**A**,**B**) and FTIR spectra (**C**) of Ti_3_AlC_2_, Ti_3_C_2_, and TiO_2_/Ti_3_C_2_.

The phase composition of as-prepared samples was analyzed by XRD pattern. It can be seen from Figure 2A that three strong diffraction peaks are found in the pattern of Ti_3_AlC_2_, with the peaks at 9.6°, 39.1° and 41.9° are well indexed to the (002), (104), and (105) lattice plane of hexagonal Ti_3_AlC_2_ (JCPDS No. 52-0875), respectively [33]. After HF etching, the characteristic (104) diffraction peak of Ti_3_AlC_2_ has thoroughly disappeared. Additionally, the (002) diffraction peak is broadened, and shift to the lower angles compared to their original location, which indicate the conversion of Ti_3_AlC_2_ to Ti_3_C_2_ [34]. After hydrothermal reaction, three new diffraction peaks are observed in the TiO_2_/Ti_3_C_2_ composite particles, with peaks at 25.3°, 37.9°, and 48.0° assigned to (101), (004), and (200) lattice plane of anatase (JCPDS no. 21-1272), respectively [35]. More importantly, the intensity of (101) diffraction peak at 25.3° markedly enhances with the increase of molar mass of TBOT (Figure 2B), which indicates that the density of TiO_2_ nanodots can be regulated with TiO_2_ precursor solution. FTIR spectra show that four obvious adsorption bands are observed in Ti_3_C_2_ and TiO_2_/Ti_3_C_2_, with 3411 cm^−1^, 3180 cm^−1^, 1635 cm^−1^, and 1322 cm^−1^ corresponding to the stretching vibration of hydroxyl group (–OH) of H_2_O, stretching vibration of Ti–OH, blending vibration of –OH of H_2_O and stretching vibration of covalent C–F bond of Ti_3_C_2_, respectively [27,36]. It is worth noting that, compared with Ti_3_C_2_, a strong adsorption band at 542 cm^−1^ is found in the spectra of TiO_2_/Ti_3_C_2_, which is assigned to the stretching vibration of Ti–O bond of TiO_2_ [37].

### 3.3. Determining the Optimal Mass Fraction of TiO_2_/Ti_3_C_2_ through Testing the Tribological Properties of 0.25 M TiO_2_/Ti_3_C_2_/Epoxy Nanocomposite

**Figure 3:**
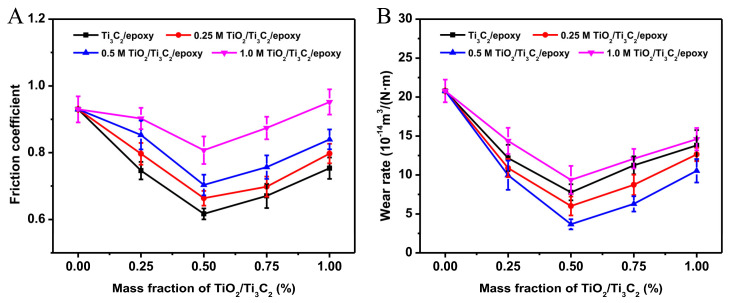
(**A**)Friction coefficient and (**B**) wear rate of TiO_2_/Ti_3_C_2_/epoxy nanocomposites with different mass friction under a normal load of 8 N.

To determine the optimal mass fraction of TiO_2_/Ti_3_C_2_ composite particles, the tribological property of TiO_2_/Ti_3_C_2_/epoxy nanocomposite with different content was investigated. As shown in Figure 3, both the friction coefficient and wear rate of all the TiO_2_/Ti_3_C_2_/epoxy nanocomposites show a trend of first decreasing and then increasing when the mass fraction of TiO_2_/Ti_3_C_2_ is increased from 0% to 1%. Among them, the TiO_2_/Ti_3_C_2_/epoxy nanocomposite with 0.5% mass fraction presents the lowest friction coefficient and minimum wear rate. Herein, the friction coefficient of TiO_2_/Ti_3_C_2_/epoxy nanocomposites at 0.5% mass fraction are determined to 0.60 for Ti_3_C_2_/epoxy, 0.66 for 0.25 M TiO_2_/Ti_3_C_2_/epoxy, 0.71 for 0.5 M TiO_2_/Ti_3_C_2_/epoxy, 0.81 for 1.0 M TiO_2_/Ti_3_C_2_/epoxy, and the wear rate of TiO_2_/Ti_3_C_2_/epoxy nanocomposites at 0.5% mass fraction are calculated to 7.66 × 10^−14^ m^3^/(N·m) for Ti_3_C_2_/epoxy, 5.86 × 10^−14^ m^3^/(N·m) for 0.25 M TiO_2_/Ti_3_C_2_/epoxy, 3.37 × 10^−14^ m^3^/(N·m) for 0.5 M TiO_2_/Ti_3_C_2_/epoxy, 9.26 × 10^−14^ m^3^/(N·m) for 1.0 M TiO_2_/Ti_3_C_2_/epoxy. Therefore, for the TiO_2_/Ti_3_C_2_/epoxy nanocomposites incorporated with different decoration density of TiO_2_/Ti_3_C_2_, the mass fraction of TiO_2_/Ti_3_C_2_ composite particles is selected as 0.5% for subsequent studies.

### 3.4. Fracture Surface Analysis of TiO_2_/Ti_3_C_2_/Epoxy Nanocomposites with Different TiO_2_/Ti_3_C_2_

**Figure 4:**
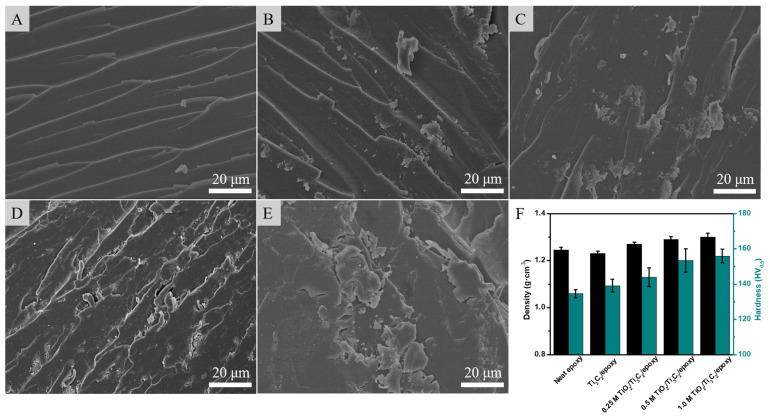
SEM images of fracture surfaces of neat epoxy (**A**), Ti_3_C_2_/epoxy (**B**), 0.25 M TiO_2_/Ti_3_C_2_/epoxy (**C**), 0.5 M TiO_2_/Ti_3_C_2_/epoxy (**D**), 1.0 M TiO_2_/Ti_3_C_2_/epoxy (**E**) nanocomposites at 0.5% mass fraction. Density and hardness (**F**) of neat epoxy and TiO_2_/Ti_3_C_2_/epoxy nanocomposites at 0.5% mass fraction.

To understand the internal microstructure of 0.5% TiO_2_/Ti_3_C_2_/epoxy nanocomposites, the morphology of fracture surfaces of TiO_2_/Ti_3_C_2_/epoxy nanocomposites incorporated with different TiO_2_/Ti_3_C_2_ was investigated. As shown in Figure 4A, large numbers of river-like stripes are clearly observed on the fracture surface of neat epoxy, which are typical characteristics of brittle thermosetting polymer [38]. For Ti_3_C_2_/epoxy nanocomposite (Figure 4B), the stripes on the fracture surface decrease, and simultaneously some loose particles are emerged on the fracture surface. After incorporation of 0.25 M TiO_2_/Ti_3_C_2_ composite particles into epoxy matrix, the stripes on the fracture surface of composite greatly decrease, but the loose particles are still found on the fracture surface (Figure 4C), which maybe implies the weak interfacial interaction between 0.25 M TiO_2_/Ti_3_C_2_ and the epoxy matrix. When incorporated with 0.5 M TiO_2_/Ti_3_C_2_, it seems that some particles are embedded in the epoxy matrix, and the loose particles can hardly be found on the fracture surface (Figure 4D), maybe ascribing to the strong interfacial interaction between 0.5 M TiO_2_/Ti_3_C_2_ and epoxy matrix. However, a large number of particles are observed on the fracture surface of 1.0 M TiO_2_/Ti_3_C_2_/epoxy nanocomposite (Figure 4E), suggesting the poor interfacial interaction between TiO_2_/Ti_3_C_2_ and matrix. In addition, the density and hardness of TiO_2_/Ti_3_C_2_/epoxy nanocomposites (0.5% mass fraction) are gradually enhanced with the increase of decorated TiO_2_ (Figure 4F), with the average density of 1.24, 1.21, 1.23, 1.29, 1.33 g·cm^–3^ and average hardness of 135, 139, 144, 153, 156 Hv0.5 for neat epoxy, Ti_3_C_2_/epoxy, 0.25 M TiO_2_/Ti_3_C_2_/epoxy, 0.5 M TiO_2_/Ti_3_C_2_/epoxy, and 1.0 M TiO_2_/Ti_3_C_2_/epoxy nanocomposites.

### 3.5. Surface Morphology of TiO_2_/Ti_3_C_2_/Epoxy Nanocomposites with Different TiO_2_/Ti_3_C_2_ before Friction Tests

**Figure 5:**
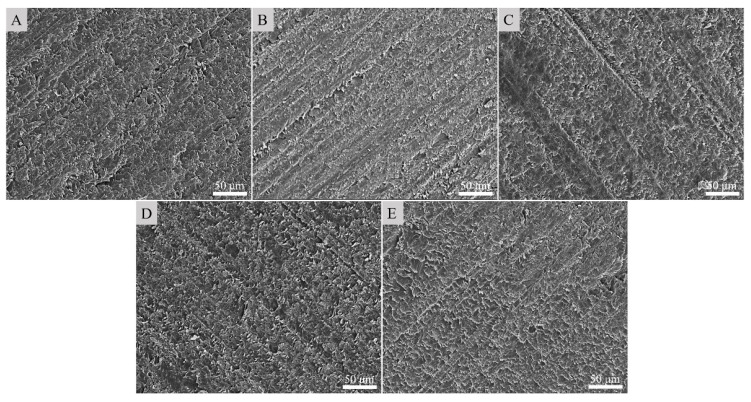
SEM images of neat epoxy (**A**), Ti_3_C_2_/epoxy (**B**), 0.25 M TiO_2_/Ti_3_C_2_/epoxy (**C**), 0.5 M TiO_2_/Ti_3_C_2_/epoxy (**D**), 1.0 M TiO_2_/Ti_3_C_2_/epoxy (**E**) nanocomposites at 0.5% mass fraction after surface polishing treatment with SiC abrasive paper.

The differences in surface irregularity of materials have an important influence on the results of friction tests. So, the surface morphology of TiO_2_/Ti_3_C_2_/epoxy samples were investigated before friction tests were carried out. As shown in Figure 5, the surface morphologies of all the samples seem to be similar, ascribing to the same treatment conditions. Additionally, the neat epoxy and TiO_2_/Ti_3_C_2_/epoxy nanocomposites display a homogeneous and regular surface morphology, without apparent protuberant or sunk part, which indicates that the effect of surface irregularity of TiO_2_/Ti_3_C_2_/epoxy nanocomposites on results of friction tests is negligible.

### 3.6. Tribological Properties of TiO_2_/Ti_3_C_2_/Epoxy Nanocomposites with Different TiO_2_/Ti_3_C_2_

**Figure 6:**
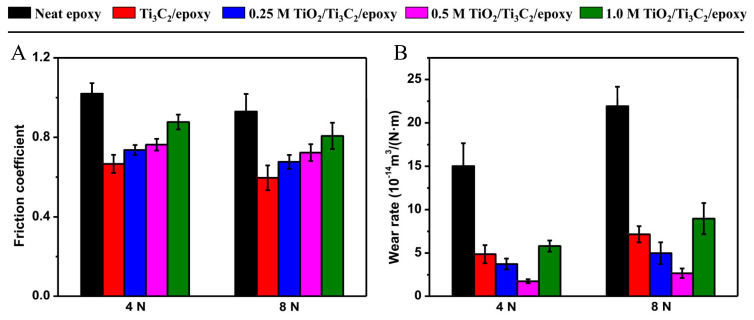
Friction coefficient (**A**) and wear rate (**B**) of neat epoxy, Ti_3_C_2_/epoxy, 0.25 M TiO_2_/Ti_3_C_2_/epoxy, 0.5 M TiO_2_/Ti_3_C_2_/epoxy, and 1.0 M TiO_2_/Ti_3_C_2_/epoxy nanocomposites at 0.5% mass fraction under the normal load of 4 N and 8 N.

The tribological property of TiO_2_/Ti_3_C_2_/epoxy nanocomposites was evaluated by measuring their friction coefficient and wear rate. As shown in Figure 6A, the neat epoxy exhibits the highest friction coefficient of 1.02 under a normal load of 4 N, and the friction coefficient dramatically decreases to 0.67 after incorporation of 0.5% Ti_3_C_2_ into epoxy matrix. When the TiO_2_/Ti_3_C_2_ composite particles were used as the fillers, the friction coefficient of TiO_2_/Ti_3_C_2_/epoxy nanocomposites increases gradually with increasing the decorated TiO_2_, and the friction coefficient of 0.25 M TiO_2_/Ti_3_C_2_/epoxy, 0.5 M TiO_2_/Ti_3_C_2_/epoxy, and 1.0 M TiO_2_/Ti_3_C_2_/epoxy are determined to 0.74, 0.76, and 0.88, respectively. Furthermore, the friction coefficient of neat epoxy, Ti_3_C_2_/epoxy, 0.25 M TiO_2_/Ti_3_C_2_/epoxy, 0.5 M TiO_2_/Ti_3_C_2_/epoxy, and 1.0 M TiO_2_/Ti_3_C_2_/epoxy under the normal load of 8 N are calculated to 0.93, 0.60, 0.68, 0.72, and 0.81, respectively, which is lower than that under the normal load of 4 N. This can be ascribed to the negative exponential relation between applied load and friction coefficient that can be expressed as μ = kN^n − 1^ [39], where the μ, k, N are the friction coefficient, constant, and applied load, respectively, and n is a constant between 2/3 and 1, depending on the amount of interaction between elastic and plastic deformation. The wear rate of neat epoxy and TiO_2_/Ti_3_C_2_/epoxy nanocomposites is shown in Figure 6B. It can be seen that the wear rate of neat epoxy is 15.02 × 10^−14^ m^3^/(N·m) under a normal load of 4 N, followed by decreases sharply to a value of 4.87 × 10^−14^ m^3^/(N·m) after incorporation of only 0.5% mass fraction of Ti_3_C_2_ microparticles. With the incorporation of 0.25 M TiO_2_/Ti_3_C_2_ into epoxy matrix, the wear rate of TiO_2_/Ti_3_C_2_/epoxy nanocomposite further decreases to 3.74 × 10^−14^ m^3^/(N·m), and gained the minimum wear rate of 1.75 × 10^−14^ m^3^/(N·m) in 0.5 M TiO_2_/Ti_3_C_2_/epoxy nanocomposite. When the normal load is enhanced to 8 N, the wear rate of all the samples is obviously increased, and calculated to 21.93 × 10^−14^ m^3^/(N·m) for neat epoxy, 7.17 × 10^−14^ m^3^/(N·m) for Ti_3_C_2_/epoxy, 4.99 × 10^−14^ m^3^/(N·m) for 0.25 M TiO_2_/Ti_3_C_2_/epoxy, 2.67 × 10^−14^ m^3^/(N·m) for 0.5 M TiO_2_/Ti_3_C_2_/epoxy and 8.97 × 10^−14^ m^3^/(N·m) for 1.0 M TiO_2_/Ti_3_C_2_/epoxy. No matter the normal load is 4 N or 8 N, 0.5 M TiO_2_/Ti_3_C_2_/epoxy nanocomposite gains the minimum wear rate, which may be attributed that the medium density TiO_2_ nanodots grown on Ti_3_C_2_ provide abundant anchors for the strong interaction between TiO_2_/Ti_3_C_2_ composite particles and epoxy matrix [40]. Besides, the wear rate of all the samples under the normal load of 8 N is always higher than that under the normal load of 4 N, mainly ascribing that the enhanced normal load produced a higher shear stress, which resulted in the detachment of more epoxy particles or fillers from the surface of epoxy nanocomposites.

### 3.7. Worn Surface Analysis of TiO_2_/Ti_3_C_2_/Epoxy Nanocomposites with Different TiO_2_/Ti_3_C_2_

**Figure 7:**
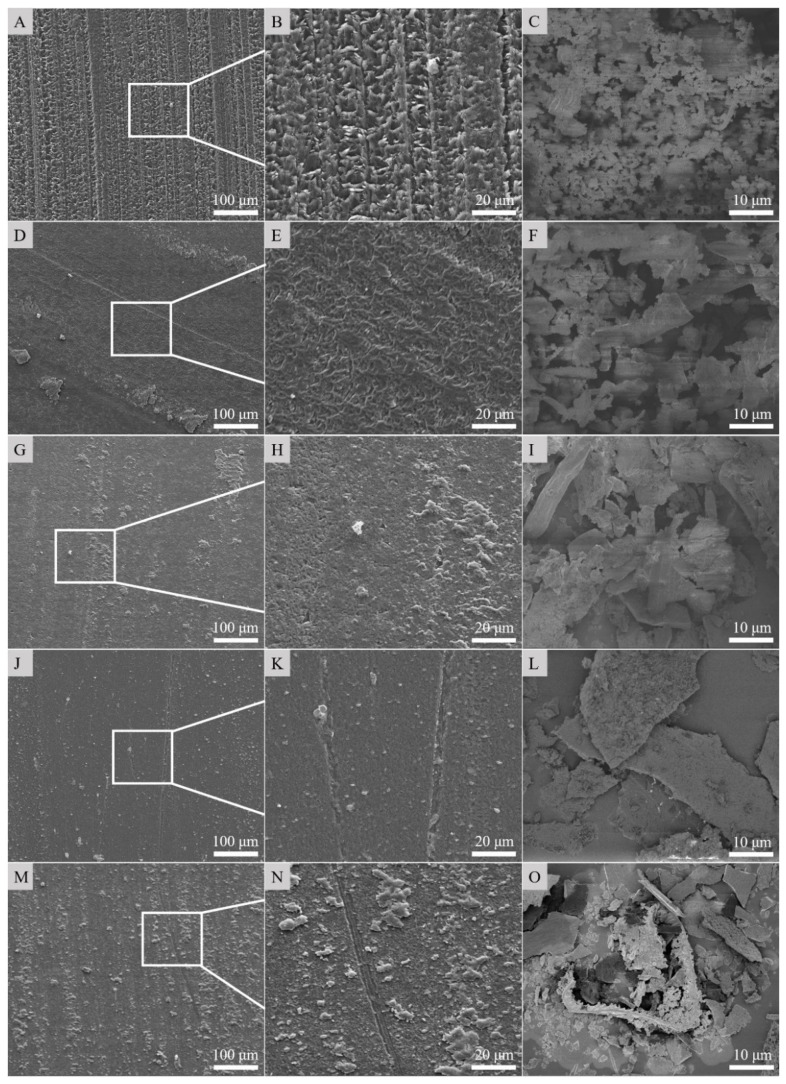
SEM images of the worn surfaces of neat epoxy (**A**,**B**), Ti_3_C_2_/epoxy (**D**,**E**), 0.25 M TiO_2_/Ti_3_C_2_/epoxy (**G**,**H**), 0.5 M TiO_2_/Ti_3_C_2_/epoxy (**J**,**K**), and 1.0 M TiO_2_/Ti_3_C_2_/epoxy (**M**,**N**) nanocomposites at 0.5% mass fraction under a normal load of 8 N. SEM images of wear debris of neat epoxy (**C**), Ti_3_C_2_/epoxy (**F**), 0.25 M TiO_2_/Ti_3_C_2_/epoxy (**I**), 0.5 M TiO_2_/Ti_3_C_2_/epoxy (**L**), and 1.0 M TiO_2_/Ti_3_C_2_/epoxy (**O**) nanocomposites at 0.5% mass fraction under a normal load of 8 N.

To understand the wear mechanism, the worn surfaces along with wear debris of neat epoxy and TiO_2_/Ti_3_C_2_/epoxy nanocomposites under the normal load of 8 N were investigated. As shown in Figure 7A,B, the neat epoxy displays a relatively rough worn surface with lots of ploughed furrows, suggesting the fatigue wear of neat epoxy, which is the typical wear form of brittle polymer [41]. During the sliding friction, the ploughed furrows can easily induce the detachment of small pieces of epoxy from the bulk epoxy under the shear stress, so large numbers of small-sized wear debris are observed on the steel counterpart surface (Figure 7C), which results in the serious wear behavior of neat epoxy. After incorporation of 0.5% Ti_3_C_2_, the amounts of surface ploughed furrows of Ti_3_C_2_/epoxy nanocomposite remarkably decrease (Figure 7D,E), and the worn surface becomes smooth. In addition, the size of wear debris of Ti_3_C_2_/epoxy nanocomposite is obviously bigger than that of neat epoxy (Figure 7F), implying the improved fatigue wear in Ti_3_C_2_/epoxy nanocomposite. For 0.25 M TiO_2_/Ti_3_C_2_/epoxy nanocomposite, the smoothness of its worn surface is further enhanced, and noticeable ploughed traces have scarcely been found (Figure 7G,H). Simultaneously, the large amounts of flake-like wear debris are collected from counterpart surface of 0.25 M TiO_2_/Ti_3_C_2_/epoxy nanocomposite (Figure 7I), which implies the improvement of wear resisting property of epoxy resin. The improved wear resisting property mainly attributed that the original brittle “epoxy to steel” contact wear is partly substituted with tough “TiO_2_/Ti_3_C_2_ to steel” contact wear [42]. When the 0.5 M TiO_2_/Ti_3_C_2_ composite particles are incorporated into epoxy matrix, the worn surface of nanocomposite becomes quite smooth (Figure 7J,K), indicating the excellent wear resisting property of 0.5 M TiO_2_/Ti_3_C_2_/epoxy nanocomposite. Additionally, some tiny particles are presented on the worn surface, which suggests that the transformation of wear form of 0.5 M TiO_2_/Ti_3_C_2_/epoxy nanocomposite from fatigue wear to abrasive wear may be made. Moreover, the flake-like wear debris with large size are collected from the counterpart surface (Figure 7L). This can be explained that, on one hand, the moderate TiO_2_ nanodots grown on TiO_2_/Ti_3_C_2_ act as the anchors to form strong mechanical interlock between 0.5 M TiO_2_/Ti_3_C_2_ and epoxy matrix, which can bear a higher normal shear stress during sliding friction; on the other hand, due to the formation of large size of flake-like wear debris, the “composite to flake-like wear debris of composite” contact wear may be established, which can reduce the wear rate of 0.5 M TiO_2_/Ti_3_C_2_/epoxy nanocomposite greatly. For the 1.0 M TiO_2_/Ti_3_C_2_/epoxy nanocomposite, a large number of tiny particles are presented on the worn surface (Figure 7M,N), and the size of wear debris of 1.0 M TiO_2_/Ti_3_C_2_/epoxy nanocomposite are much smaller than that of 0.5 M TiO_2_/Ti_3_C_2_/epoxy nanocomposite (Figure 7O), which reveals that the dominant wear form of 1.0 M TiO_2_/Ti_3_C_2_/epoxy nanocomposite is abrasive wear, instead of fatigue wear. These results demonstrated that the micromorphology of TiO_2_/Ti_3_C_2_ composite particles can strongly influence the wear behavior of epoxy nanocomposites.

### 3.8. Dynamic Mechanical Analysis of Ti_3_C_2_/Epoxy Nanocomposites with Different TiO_2_/Ti_3_C_2_

**Figure 8:**
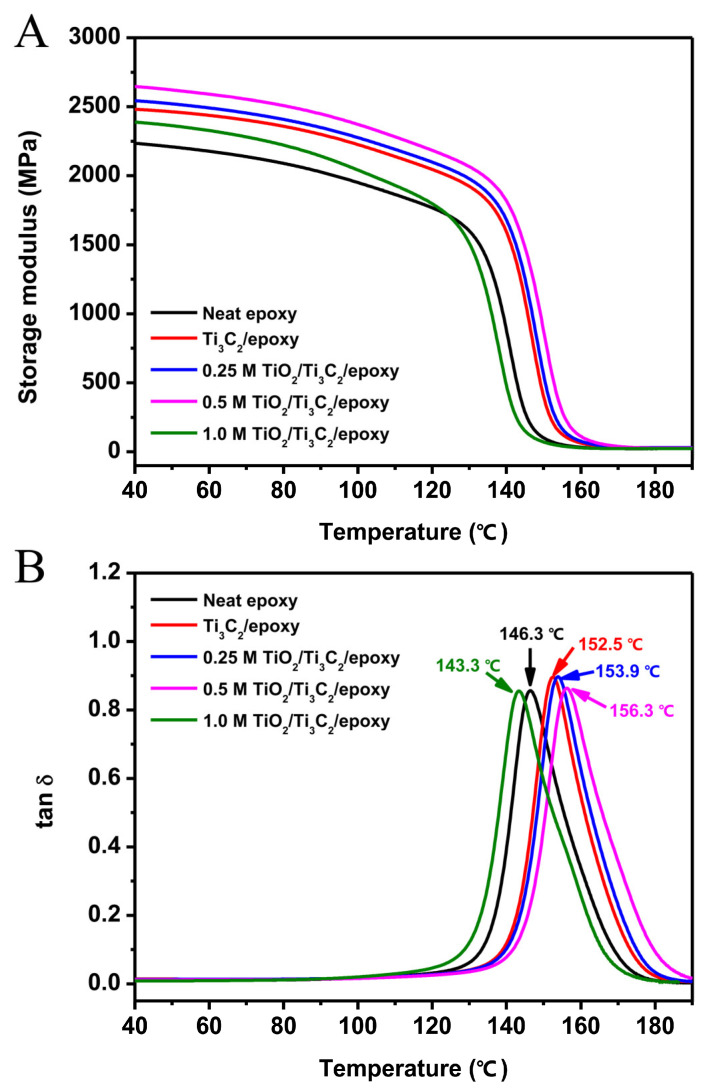
Storage modulus (**A**) and tan δ (**B**) curves versus temperature for neat epoxy and TiO_2_/Ti_3_C_2_/epoxy nanocomposites.

To further analyze the thermo-mechanical properties of neat epoxy and TiO_2_/Ti_3_C_2_/epoxy nanocomposites, dynamic mechanical analysis was performed. It can be seen from Figure 8A that the storage modulus of TiO_2_/Ti_3_C_2_/epoxy nanocomposites in the glassy state is gradually enhanced with the increase of decorated TiO_2_ nanodots of TiO_2_/Ti_3_C_2_ composite particles, followed by reach to the maximum value at 0.5 M TiO_2_/Ti_3_C_2_/epoxy nanocomposite and decrease in 1.0 M TiO_2_/Ti_3_C_2_/epoxy nanocomposite. Herein, the storage modulus of Ti_3_C_2_, 0.25 M TiO_2_/Ti_3_C_2_/epoxy, 0.5 M TiO_2_/Ti_3_C_2_/epoxy, and 1.0 M TiO_2_/Ti_3_C_2_/epoxy at 40 °C are determined to 2481.3 MPa, 2543.4 MPa, 2645.9 MPa, 2388.5 MPa, which is increased by 11.0%, 13.8%, 18.4%, and 6.9%, respectively, compared with neat epoxy (2235.2 MPa at 40 °C). As shown in Figure 8B, the Tg of all the samples show a similar trend of raising first and then falling, as observed in their storage modulus, with the Tg of neat epoxy, Ti_3_C_2_/epoxy, 0.25 M TiO_2_/Ti_3_C_2_/epoxy, 0.5 M TiO_2_/Ti_3_C_2_/epoxy, and 1.0 M TiO_2_/Ti_3_C_2_/epoxy are determined to 146.3 °C, 152.5 °C, 153.9 °C, 156.3 °C, and 143.3 °C, respectively. The increased storage modulus and Tg can be attributed that, on one hand, the TiO_2_ nanodot protuberances maybe provided the anchors to form the mechanical interlock between TiO_2_/Ti_3_C_2_ and epoxy matrix, which physically hindered the molecular mobility of epoxy matrix [43]; on the other hand, the enhanced contact area between TiO_2_/Ti_3_C_2_ and epoxy resulting from TiO_2_ nanodots, as well as abundant functional groups (–OH, –F) on TiO_2_ and Ti_3_C_2_ surface induced a strong interaction between TiO_2_/Ti_3_C_2_ and epoxy matrix, which restricted the molecular motion of the local epoxy around TiO_2_/Ti_3_C_2_ [44]. The minimum Tg in 1.0 M TiO_2_/Ti_3_C_2_/epoxy nanocomposites maybe ascribed to the poor interaction between 1.0 M TiO_2_/Ti_3_C_2_ and epoxy matrix, as well as the reduced degree of cross-linking reaction of epoxy resin resulting from the high-density TiO_2_ nanodots of 1M TiO_2_/Ti_3_C_2_ [45].

### 3.9. A Proposed Mechanism for the Improved Tribological and Thermo-Mechanical Properties of TiO_2_/Ti_3_C_2_/Epoxy Nanocomposites

**Scheme 2:**
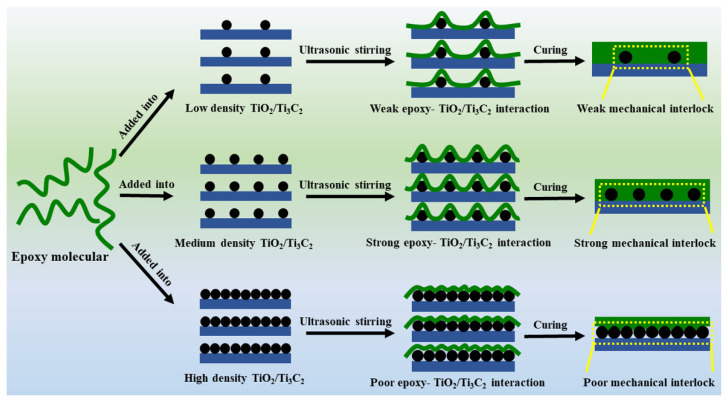
Illustration for the interaction between TiO_2_/Ti_3_C_2_ and epoxy matrix in TiO_2_/Ti_3_C_2_/epoxy nanocomposites.

On the basis of the abovementioned results, a plausible mechanism is proposed for shedding light on the improved tribological and thermo-mechanical properties of TiO_2_/Ti_3_C_2_/epoxy nanocomposite, as illustrated in Scheme 2. For the low density TiO_2_/Ti_3_C_2_ incorporated epoxy nanocomposite, the sparse TiO_2_ nanodots grown on Ti_3_C_2_ surface only provide a small amount of TiO_2_ nanodot anchors, which induces a weak interfacial interaction and mechanical interlock between TiO_2_/Ti_3_C_2_ and epoxy matrix. As a result, the tribological and thermo-mechanical properties of TiO_2_/Ti_3_C_2_/epoxy nanocomposite are enhanced to some extent. When the medium density TiO_2_/Ti_3_C_2_ are used as fillers, the moderate TiO_2_ nanodots provide lots of effective anchors (suitable gap between adjacent anchors), supporting the strong interfacial interaction and mechanical interlock between TiO_2_/Ti_3_C_2_ and epoxy matrix. Therefore, the tribological and thermo-mechanical properties of TiO_2_/Ti_3_C_2_/epoxy nanocomposite are greatly enhanced. However, the dense TiO_2_ nanodots hardly provide effective anchors for the TiO_2_/Ti_3_C_2_-epoxy interfacial interaction, leads to a poor mechanical interlock between TiO_2_/Ti_3_C_2_ and epoxy matrix. Thus, the worse tribological and thermo-mechanical properties are gained in the high density TiO_2_/Ti_3_C_2_ incorporated epoxy nanocomposite.

## 4. Conclusions

In summary, the TiO_2_ decorated Ti_3_C_2_ were synthesized by hydrothermal growth of TiO_2_ nanodots onto the surface of accordion-like Ti_3_C_2_, and the different decoration degree (low, medium, high density) of TiO_2_/Ti_3_C_2_ composite particles were prepared by regulating the concentration of TiO_2_ precursor solution. Tribological test results indicated that the incorporation of TiO_2_/Ti_3_C_2_ can effectively improve the wear rate of epoxy resin, and the improvement effect was dependent on the density of TiO_2_ nanodots of TiO_2_/Ti_3_C_2_. Among, the medium density TiO_2_/Ti_3_C_2_ could improve the wear rate of epoxy resin at the greatest extent, with a wear rate of only 2.67 × 10^−14^ m^3^/(N·m) was obtained under the normal load of 8 N. This mainly ascribed that the moderate TiO_2_ nanodot protuberances on the Ti_3_C_2_ surface induced a strong mechanical interlocking effect between medium density TiO_2_/Ti_3_C_2_ and epoxy matrix, which can bear a higher normal shear stress during sliding friction. The morphologies of worn surfaces and wear debris revealed that the wear form was gradually transformed from abrasive wear in neat epoxy to adhesive wear in 1.0 M TiO_2_/Ti_3_C_2_/epoxy nanocomposites. Moreover, the results of thermo-mechanical property indicated that incorporation of TiO_2_/Ti_3_C_2_ also effectively improved the storage modulus and glass transition temperature of epoxy resin. Our results demonstrated that the tribological and thermo-mechanical properties of TiO_2_/Ti_3_C_2_/epoxy nanocomposites can be strongly influenced by the micromorphology of TiO_2_/Ti_3_C_2_, which provides a promising strategy for improving the tribological and thermo-mechanical properties of epoxy nanocomposites.

## Data Availability

The data presented in this study are available on request from the corresponding author.
